# High Temperature Mechanical Response and Failure Analysis of 3D Five-Directional Braided Composites with Different Braiding Angles

**DOI:** 10.3390/ma12213506

**Published:** 2019-10-25

**Authors:** Hong-mei Zuo, Dian-sen Li, Lei Jiang

**Affiliations:** 1 Key Laboratory of Bio-Inspired Smart Interfacial Science and Technology, Ministry of Education, School of Chemistry, Beihang University, Beijing 100191, China; hongmeizuo@buaa.edu.cn (H.-m.Z.); jianglei@buaa.edu.cn (L.J.); 2 Beijing Advanced Innovation Center for Biomedical Engineering, Beihang University, Beijing 100191, China

**Keywords:** 3D braided composites, five-directional braiding, high-temperature properties, failure mechanism

## Abstract

Three-dimensional (3D) five-directional braided composites are extensively applied in aeronautics and national defense due to their integrity and structural superiorities. In this paper, 3D five-directional braided carbon/epoxy composites were manufactured, and the high temperature mechanical response and failure mechanisms of composites with braiding angles of 21° and 32° were studied. The out-of-plane compression tests of composites with different braiding angles were conducted at temperatures ranging from 25 °C to 180 °C. Then compression stress–strain curves, compression mechanical response, and failure modes of composites at high temperatures were analyzed and compared. The results show that compression stress–strain curves linearly increased at the initial stage and dropped at various degrees at different temperatures for composites with different braiding angles. The temperature and braiding angle were both important parameters affecting out-of-plane compression properties of 3D five-directional braided composites. Mechanical properties decreased with increasing temperature for both 21° and 32° specimens. Moreover, composites with a small braiding angle possessed higher properties at each temperature point. The morphologies manifested that the failures were a symmetric ±45° shear crack for 21° specimens and a thorough 45° shear crack for 32° specimens, and a 45° fracture weakened with increasing temperature.

## 1. Introduction

Three-dimensional (3D) multi-directional braided composites have attracted great attention owning to their unique integrity structure, outstanding designability characteristics, and excellent mechanical properties, such as high damage tolerance, high impact damage tolerance, fatigue resistance, etc. [1]. 

Many researches on mechanical properties and failure mechanism of 3D four-directional braided composites have been carried out. Tang et al. [2] investigated dynamic damage and failure mechanism of 3D braided carbon fiber/epoxy composites. Li et al. [3,4] studied the compression behaviors and failure mechanism of 3D four-directional braided composites. Pan et al. [5] studied the high strain-rate properties (1300 s^−1^ to 2100 s^−1^) of 3D braided composite material in the cryogenic field (26 °C, −50 °C, −100 °C, and −140 °C). Fan et al. [6] researched the thermo-oxidation stability of 3D braided carbon/epoxy composites. Zheng et al. [7] tested the effects of braided architecture on the tensile properties of carbon–aramid hybrid 3D braided composites. Zhou et al. [8] reported the damage mechanisms in 3D circular braided composite tubes under transverse impact. Zhang et al. [9] analyzed damage failure mode of 3D four braided composites under flexural load. Liu et al. [10] studied damage and failure mechanism of 3D carbon fiber/epoxy braided composites after thermo-oxidative ageing under transverse impact compression.

There are limited researches on 3D five-directional braided composites. Li et al. [11,12] investigated the longitudinal and transverse compressive behaviors of 3D five-directional carbon/phenolic braided composites at high strain rates and analyzed their failure mechanism. Gao et al. [13] conducted experimental modal analysis of 3D five-directional cantilever beams. Lu et al. [14] studied the tensile properties of 3D full five-directional braided composites. Guo et al. [15] presented an analytical method for designing a 3D five-directional braided composite joint. Sahoo et al. [16] evaluated elastic properties of 3D full-directional properties. Yan et al. [17] researched low-velocity impact behaviors of 3D five-directional carbon/epoxy braided composite panels with different braiding parameters. Zhang [18] analyzed meso-scale progressive damage of 3D five-directional braided composites with a braiding angle of 20°, 30°, and 45° based on the finite element model, which indicated that transverse compression strength was more relevant with the matrix properties than the braiding angle. Hu et al. [19] and Wang et al. [20] analyzed bending properties and bearing abilities of 3D full five-directional braided composites, respectively. Ouyang et al. [21] carried out experiments on the bending fatigue behaviors of 3D five-directional braided T-shaped composites. However, the high temperature mechanical response of 3D five-directional braided composites with different braiding angles has not been reported. Mei et al. [22] investigated the effect of carrier configuration on a 3D full five-directional rotary braided structure. To our knowledge, the high temperature mechanical properties and failure mechanism of 3D five-directional braided composites has not been reported.

In this article, 3D five-directional braided carbon/epoxy composites with different braiding angles were successfully elaborated. Out-of-plane compression stress–strain curves were plotted based on experimental data, compression strength and modulus at different temperatures were analyzed, and fracture morphologies and failure mechanism were simultaneously studied and compared. As for 3D five-directional braided composites, this paper sets up a detailed data system for future investigation of out-of-plane compression properties at different temperatures for either experimental or simulation studies.

## 2. Materials and Methods 

### 2.1. Materials

**Figure 1:**
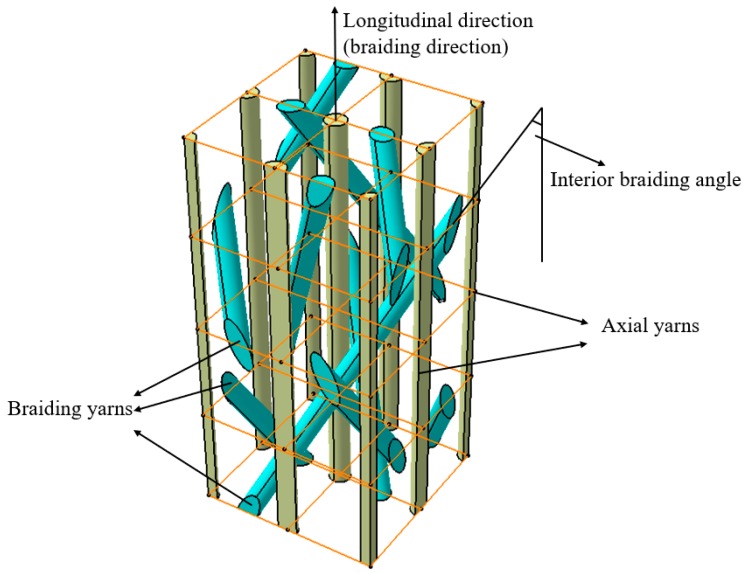
Interior architecture of three-dimensional (3D) five-directional braided composites.

**Figure 2:**
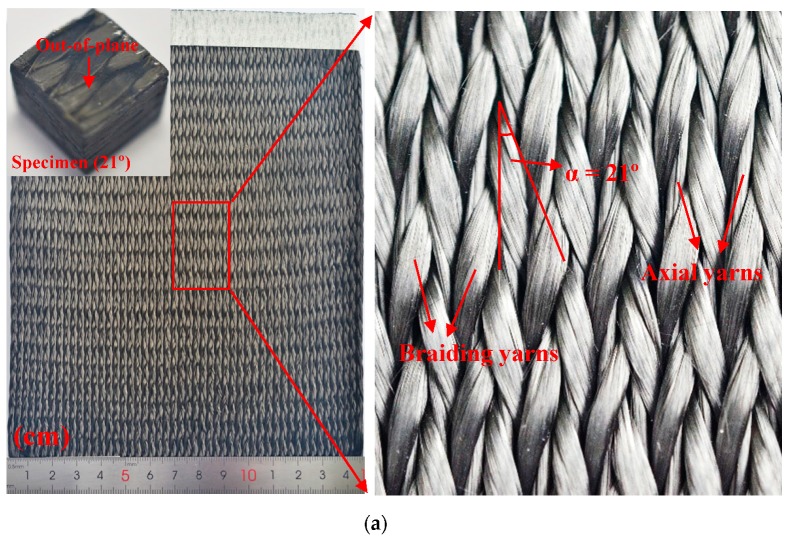

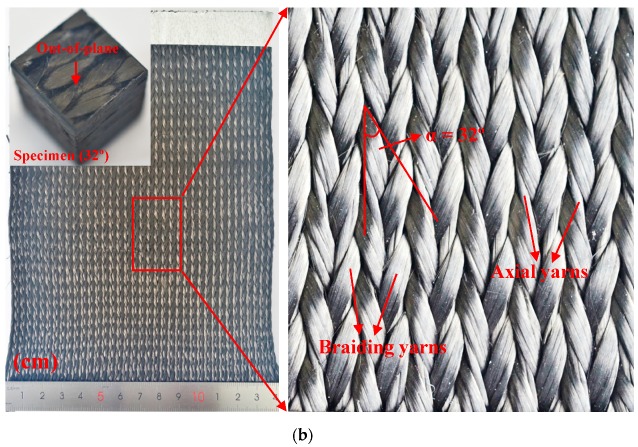
3D five-directional braided preforms and composites. (**a**) Preform with 21° braiding angle and compression specimen. (**b**) Preform with 32° braiding angle and compression specimen.

3D five-directional braided preforms were manufactured with T300-3K carbon yarns (tensile strength, 3200 MPa; tensile modulus, 230 GPa; elongation, 1.5%; density, 1.76g·cm^−3^) by a four-step 1 × 1 braiding technique [23]. The braiding process is controlled by carrying braiding yarn movement using the carrier, and braiding yarns are interwoven with each other. The yarn carrier moves along a fixed orbit, and braiding yarns on the carrier correspondingly move along the direction of the carrier. The movement of braiding yarn is always half a step behind that of the carrier. The axial yarn only moves in the direction of a line and does not participate in weaving, which is evenly sandwiched between braiding yarns. The braiding angles of 3D five-directional composites are controlled by pitch height during the braiding process. Figure 1 shows the fiber architecture of the interior unit of the 3D five-directional braided composites. The photographs of preforms with different braiding angles (21°, 32°) are shown in Figure 2.

TDE-90 epoxy resin (4,5-epoxycyclohexane-1,2-dicarboxylic acid diglycidyl ester, epoxy value = 0.90 ± 0.02 mol/100 g, viscosity = 2~5 Pa·s at 25 °C) was used as the matrix, and supplied by Tianjin Jingdong Chemical Composites Ltd (Tianjin, China). m-Phenylenediamine was purchased from Shanghai Macklin Biochemical Co., Ltd. (Shanghai, China) and used as curing agent.

Epoxy resin of TDE-90 and the curing agent were mixed and then injected into the preforms using the vacuum assisted resin transfer molding (VARTM) technique. The curing process followed a stepwise procedure of 85 °C for 2 h and 150 °C for 2 h. The braided composites were cut as Figure 2 illustrates. The information of the specimens is detailed in Table 1.

### 2.2. Experimental Procedure

**Figure 3:**
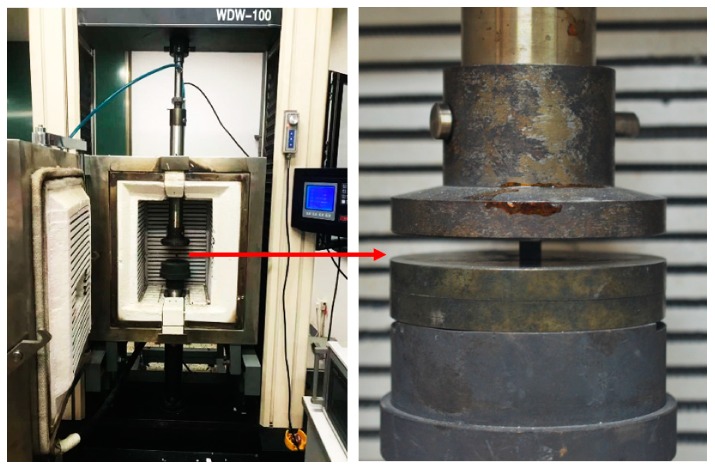
High temperature compression experiment setup.

**Figure 4:**
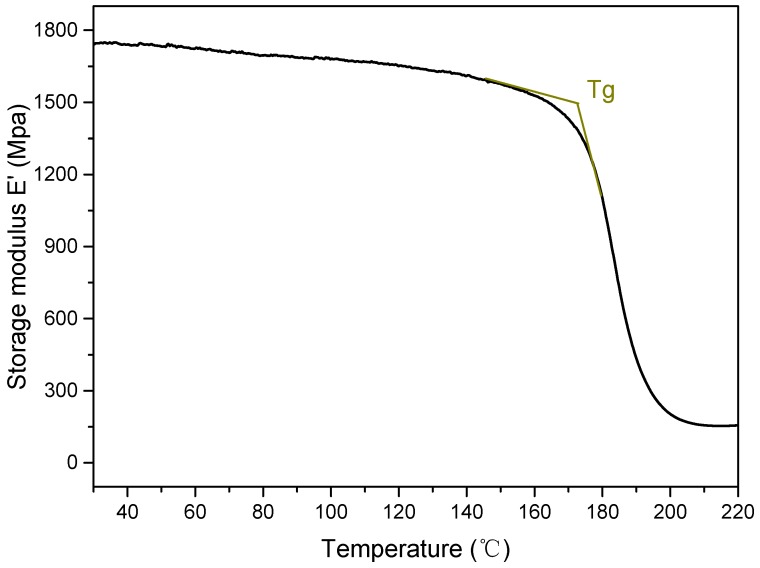
Dynamic mechanical analysis (DMA) temperature sweep results of epoxy resin.

The compression tests were conducted by a high temperature mechanical machine (Figure 3) following the HB 7571-1997 standard [24]. The compressive speed was set as 0.5 mm/min. Three specimens were tested at the same experimental condition. To investigate the effect of temperature on compression properties of the composites, the glass transition temperatures (T_g_) of the epoxy matrix were measured by dynamic mechanical analysis (DMA). Since the T_g_ of the epoxy matrix is 172 °C as shown in Figure 4, the highest tested temperature point was set as 180 °C. In this case, six temperature points (25 °C, 60 °C, 90 °C, 120 °C, 150 °C, and 180 °C) were selected to explore the temperature effects on compression properties of the specimens.

### 2.3. Characterization

A dynamic mechanical analyzer (DMA, NETZSCH, Selb, Germany) was used to verify T_g_ of the epoxy matrix. The morphologies of the damaged composites were observed with cameras and scanning electron microscopy (SEM, Quanta 250 FEG, Plzen, Czech Republic).

## 3. Results and Discussion

### 3.1. Compression Stress–Strain Curves of Composites

**Figure 5:**
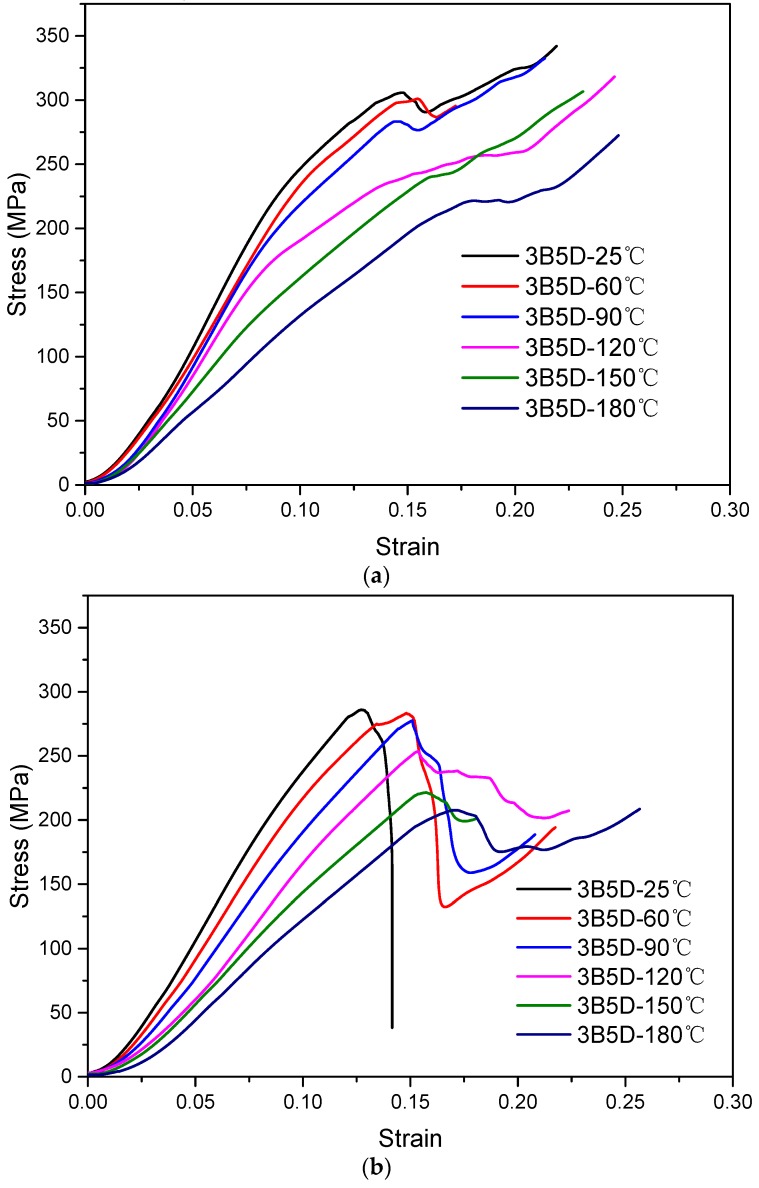
Compression stress versus strain curves of composites at different temperatures. (**a**) Specimens with braiding angle of 21°. (**b**) Specimens with braiding angle of 32°.

The out-of-plane compression stress versus strain curves of specimens with 21° braiding angle at different temperatures are shown in Figure 5a. It was found that the slopes of the curves decrease with increasing temperature at the initial stage. At 25 °C, the curve undergoes the initial elastic stage and the yielding stage; stress increases linearly at the elastic stage and then rises slowly until stress drops with a buffer during which it reaches the maximum value. The curve trend is in accordance with those from other literature [25]. Soon afterwards, the stress begins to increase again. The curve transition from linear to nonlinear indicates that the internal crack is growing slowly, and the appearance of the buffer indicates that the crack growth is greatly stropped by the axial yarns; the load rising again after stress peak manifests that the fiber bundles are further compacted in the thickness direction. At 60 °C and 90 °C, the curves change similarly with that at 25 °C, and they increase linearly at the initial stage, increase slowly with decreasing slopes, then they immediately drop a little, and finally they begin to rise again. At 120 °C, the nonlinear growth segment is very long, showing that the plastic features begin to occur at earlier times. In addition, the loading drop is not obvious. This might be because the epoxy matrix becomes soft and starts to lose its mechanical properties [26]. At 150 °C and 180 °C, the curve slopes decrease significantly with the increase of temperature because the epoxy matrix is softer and the interface between fibers and matrix does not bond tightly anymore. At the same time, the plastic characteristic of the matrix becomes more obvious before the specimens are destroyed.

Figure 5b shows the compression stress versus strain curves of the specimens with large braiding angle, 32°, at different temperatures. At 25 °C, the curve shows linearity and the stress immediately drops when it reaches the maximum value, indicating a brittle fracture feature. This is because the mechanical properties are dominated by fiber bundles and matrix together for large braiding angle specimens, and the brittle fracture of matrix plays a large role at a low temperature. At 60 °C and 90 °C, the curves increase linearly at the initial stage, then they drop with a higher magnitude, and finally they begin to rise again. At 120 °C, the stress drops gradually after the maximum stress point because the matrix begins to soften. At 150 °C and 180 °C, the curve slopes decline obviously because the matrix softens and the interface bond between fibers and matrix is no longer tight. Meanwhile, the stress decreases in a little magnitude after the maximum value when the stress increases again owing to the more obvious plastic feature.

For both composites with different braiding angles, the curves decline when the temperature rises. This could be explained by the matrix being softened and plasticized step by step, and the outstanding mechanical properties of the matrix which gradually decline at a lower temperature. Moreover, the stress–strain curves of small braiding angle specimens are superior to large braiding angle specimens at each temperature point. This is because the mechanical property of composites with a small braiding angle is mainly determined by fibers; whereas for a large braiding angle composite the property is determined by fibers and matrix together. In addition, the stress of a specimen with a 21° braiding angle decreases by a very small magnitude compared to that of a 32° braiding angle after peak stress. This is because the specimen with a small braiding angle is more tightly woven than that of a 32° specimen, and there is less space to be compacted. Afterwards, the compacted fiber bundles begin to bear loading again. 

### 3.2. Out-of-Plane Compression Strength and Modulus 

**Figure 6:**
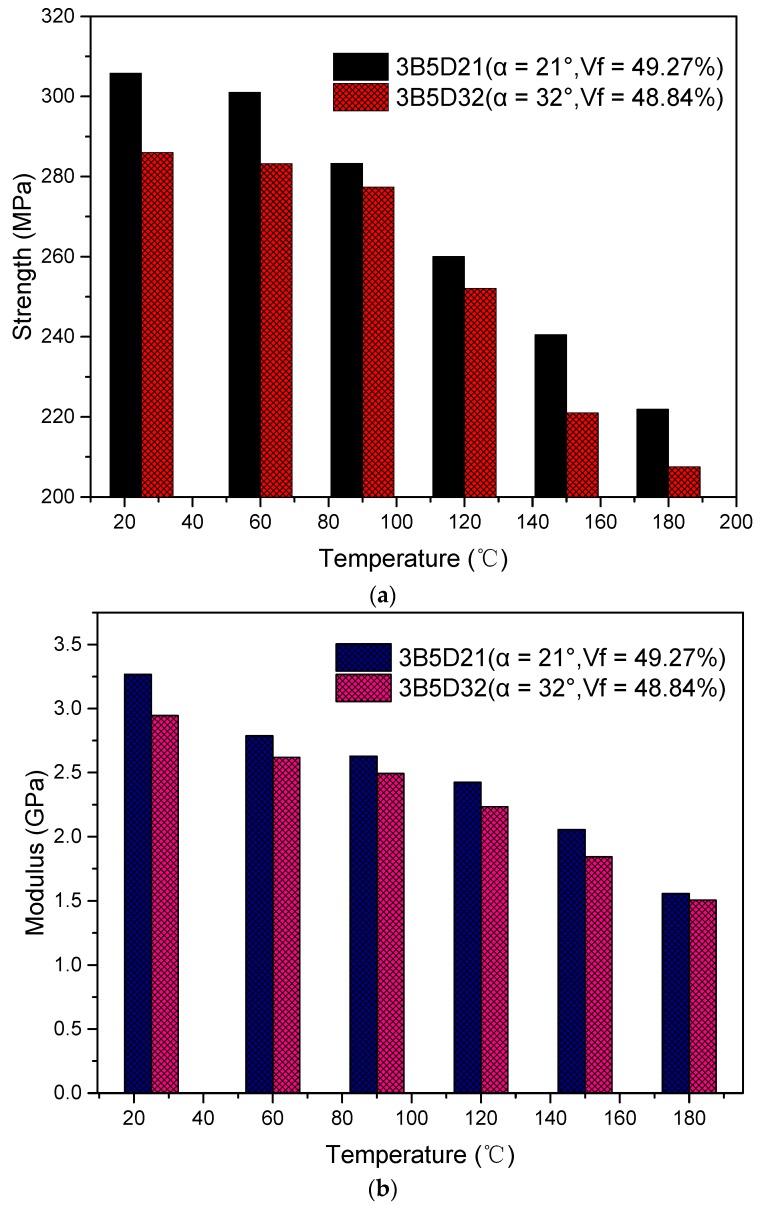
The out-of-plane compression properties at different temperatures. (**a**) Compression strength; (**b**) compression modulus.

Figure 6a and Table 2 show the compression strength of specimens with a braiding angle of 21° and 32° at different temperatures. The compression strength decreases with increasing temperature for the two kinds of composites. The strength value at 25 °C is improved by 34.72% compared to 3D four-directional composites with a 20° braiding angle of Huang’s work and 91.13% of Zhang’s work, respectively, as indicated in previous studies [25,27]. This further proves that by adding fifth axial yarns, compression resistance of 3D five-directional composites is superior to that of 3D four-directional composites. From 60 °C to 180 °C, the strength comparatively decreases by 1.56%, 7.36%, 14.98%, 21.36%, 27.44% and 0.96%, 3.01%, 11.84%, 22.72%, 37.77% when comparing with that at 25 °C. This is because the mechanical properties of the matrix decrease with the temperature increasing and the matrix shrinkage phenomenon begins to take place, thus the matrix cannot tightly bond with the fibers.

Figure 6b and Table 2 show that the compression modulus of specimens with braiding angles of 21° and 32° at different temperatures. The compression modulus decreases with rising temperature for the two kinds of composites. The modulus for 21° specimen increased by 15.66% more than that of a 3D four-directional composite at 25 °C, as shown in previous work [28]. The main reason is because the added axial yarns also act as additional load-bearing objects. At the same time, the compression modulus for a 32° specimen improved by 4.63% compared to that of a 3D five-directional composite with a 30° braiding angle at 25 °C [29]. From 60 °C to 180 °C, the modulus comparatively decreases by 14.66%, 19.54%, 25.78%, 37.05%, 52.36% and 11.06%, 15.36%, 24.16%, 37.44%, 48.83% than that at 25 °C, respectively.

Both strength and modulus decrease with the increasing temperature for two braiding angle composites. This indicates that the mechanical resistance of a 3D five-directional braided carbon/epoxy composite to high temperature is dependent on a softening matrix and matrix decomposition [30,31]. In addition, molecular motion accelerates with rising temperature in the composites [32]. Another reason is the different thermal expansion coefficient (CTE) between carbon fiber and resin matrix. The CTE of carbon fiber is −1.0 × 10^−6^/°C in the longitudinal direction, 12.0 × 10^−6^/°C in the transverse direction [33], and TDE-90 is 31.7 × 10^−6^/°C [34]. The increasing temperature causes matrix shrinkage and further induces interfacial debonding especially at 180 °C [27]. Meanwhile, carbon fibers are inert to temperature below 200 °C and the failure of specimens can be generally interpreted by matrix degradation [35]. The compression strength and modulus of the specimens with a large braiding angle are smaller than those with a small braiding angle at each temperature point. They diminish by 6.95%, 6.30%, 2.15%, 3.15%, 8.84%, 6.91% and 10.88%, 6.39%, 5.40%, 8.52%, 11.57%, 3.22% at each temperature point, respectively. This could be because the mechanical properties of small braiding angle specimens are carried more by the fibers; whereas in large braid angle specimens, both fibers and resin dominate the mechanical properties. Meanwhile, the mechanical properties of fibers are much higher than that of matrix.

### 3.3. The Damage and Failure Mechanism

**Figure 7:**
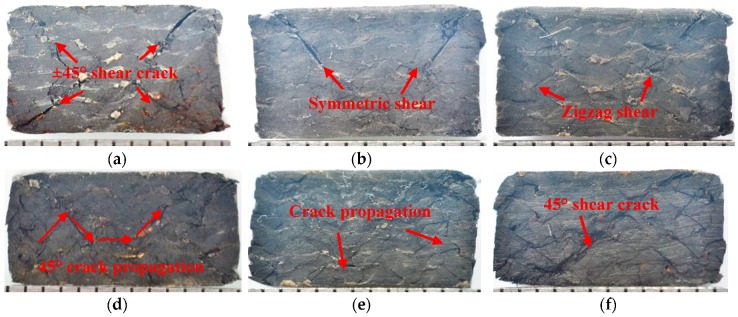
Damage morphologies of specimens with 21° braiding angle at different temperatures: (**a**) 25 °C, (**b**) 60 °C, (**c**) 90 °C, (**d**) 120 °C, (**e**) 150 °C, and (**f**) 180 °C.

Figure 7 shows the damage morphologies of 21° specimens tested at different temperatures. No obvious structural damage is observed on out-of-plane surface after compression and the structure failure mainly occurred in the longitudinal direction. From 25 °C to 90 °C, ±45° diagonal shear damage was obvious, as was symmetric shear damage due to stress mainly carried by fibers for small braiding angle composite. The added fifth component of axial yarns inhibits the stress drastically transmitting through the 45° shear crack, and symmetric failure mode could prevent the specimen from being destroyed thoroughly at the same angle, compared with that of 3D four-directional composites [4]. Moreover, the 45° shear crack becomes gradually obscure with increasing temperature since the stress is not effectively transmitted to fibers owning to the decrease in mechanical performance of the softening matrix. The discontinuous transmission of stress between fibers results in the 45° crack becoming smaller. At 25 °C, 45° shear takes place in four corners; at 60 °C and 90 °C, 45° shear takes place in two corners. The cracks of 60 °C are longer and more serious than at 90 °C. This is because as the temperature increases, the decline of mechanical properties for the epoxy matrix gives rise to fiber detaching from each other before the stress is transmitted to the next fiber bundles. At 90 °C, zigzag shear cracks emerge along the direction of braiding yarns due to the softening of the matrix. Another obvious feature is matrix extrusion; this is because the bent braiding yarns and axial yarns are compressed straight or broken and the matrix is further squeezed out. From 120 °C to 180 °C, the matrix extrusion phenomenon is not as serious as that (25–90 °C), however, the thickness of the specimens is squashed in a great magnitude. The two features could be explained by matrix plasticizing; the stress could not be transmitted to the fibers, thus dispersing over the matrix or along fiber bundles. At 120 °C and 150 °C, a small 45° crack propagation, loose fiber bundles, and matrix softness results in structure deformation. In addition, axial yarns prompt the fiber bundles to deform at a small angle instead of a large angle. At 180 °C, the specimen is more seriously broken and has significant damage deformation with a long 45° shear crack along the thickness direction, and there are also some small cracks along the fiber bundles. The reason is that the matrix is softer, and the plasticizing and decomposition of the matrix leads to mechanical performance loss; this is severe when the temperature is above T_g_ and the specimen cannot absorb stress because the fiber bundles have loosened and axial yarns cannot effectively stop the fiber bundles from deforming at a large angle; thus, a large 45° shear crack and small cracks occur.

**Figure 8:**
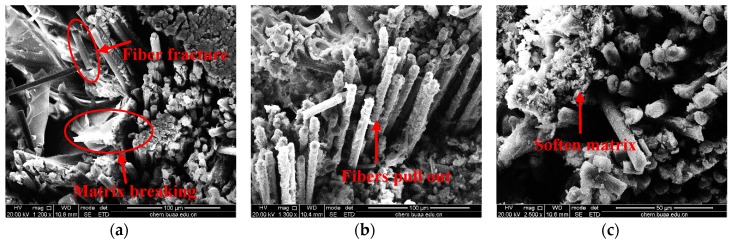

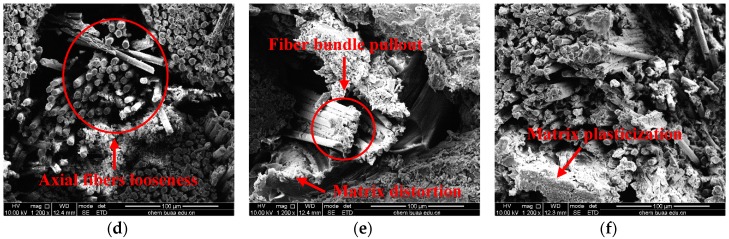
Scanning electron microscopy (SEM) photographs of specimens with 21° braiding angle at different temperatures: (**a**) 25 °C, (**b**) 60 °C, (**c**) 90 °C, (**d**) 120 °C, (**e**) 150 °C, and (**f**) 180 °C.

SEM photographs further show the damage and breakage of 21° specimens at different temperatures in Figure 8. At 25 °C, the specimen damage comes from crack propagation due to fiber fractures and matrix breaking, while the axial yarns effectively inhibit the spread of cracks under compression loading. Moreover, interface between fibers and matrix maintains high bonding. At 60 °C, the fiber pullout and interface debonding is more obvious, while the fibers still possess good bonding with the matrix. At 90 °C, the matrix softens. It can be observed that there is no obvious fiber bundles pullout, and axial yarns still restrain the braiding yarns. At 120 °C, fiber bundle deformation becomes serious, and the axial yarns become loose due to the softening matrix which cannot bond well with the fibers. At 150 °C, the fiber and matrix interface becomes more severe and the matrix distortion is evident. The fiber bundles are pulled out integrally before the stress is transmitted to each single fiber due to the matrix plasticizing. At 180 °C, fibers lose their mechanical properties because the matrix is thoroughly plasticized, and matrix decomposition occurs when the temperature of the epoxy matrix is over T_g_ (172 °C).

**Figure 9:**
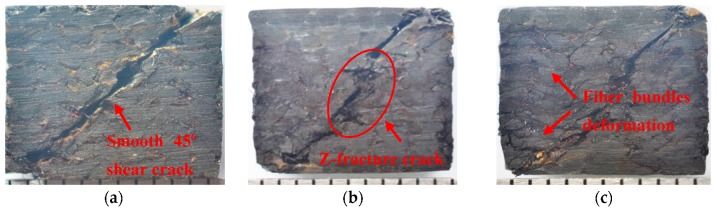

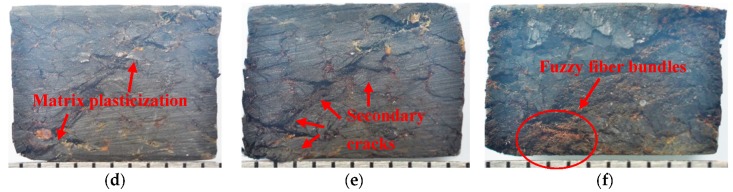
Damage morphologies of specimens with 32° braiding angle at different temperatures: (**a**) 25 °C, (**b**) 60 °C, (**c**) 90 °C, (**d**) 120 °C, (**e**) 150 °C, and (**f**) 180 °C.

With regard to 32° specimens, their damage morphologies at different temperatures are shown in Figure 9. Like 21° specimens, the damage of 32° specimens also mainly occurs in the thickness direction. The damage is more severe, and the 45° shear crack propagates through the whole specimen since the stress of 32° specimens is carried by fibers and matrix together, whereas in 21° specimens the stress is carried only by the fibers. At 25 °C, the loud sound appears because the composites exhibit brittle damage at room temperature. Matrix cracks and fibers breaking result in the specimen being finally broken, and 45° shear failure from the direction of stress force brings about a large smooth crack. In addition, the specimen easily divides into two parts along the long crack. The structure is more likely to become destructed than the 21° specimen. At 60 °C, a Z-fracture shear crack along the long 45° shear crack is obvious, when the 45° crack propagates, the axial yarns act as an obstruction and lead to the formation of secondary cracks in the thickness direction. Moreover, this specimen is also easy to divide into two parts along the long crack and the damage is also more serious than that of 21° specimen since it exhibits more brittle features for a large braiding angle composite. At 90 °C, fiber bundles deformation diffuses the stress concentration along the 45° shear crack, and the long crack is less obvious due to the matrix softening. However, the fatal crack is still longer than that of the 21° specimen. At 120 °C and 150 °C, fiber bundles deformation becomes increasingly obvious, and small cracks appear around the fiber bundles due to the weak bonding between the fibers and matrix. At the same time, secondary cracks along the long 45° shear crack are observed due to axial yarns playing a role in preventing the long crack from propagating. In addition, and much like the 21° specimen, the plasticity and softening of the matrix lead to the specimens becoming more easily compacted and the thickness becomes thinner in a great magnitude when compared to other specimens. At 180 °C, the 45° shear crack along the diagonal is similarly observed in the 21° specimen. Nevertheless, it can be seen that the structure of the fiber bundles is destroyed due to further plasticization and decomposition of the matrix when the temperature is above T_g_, and the boundary between fiber bundles of braiding yarns and axial yarns becomes fuzzy.

**Figure 10:**
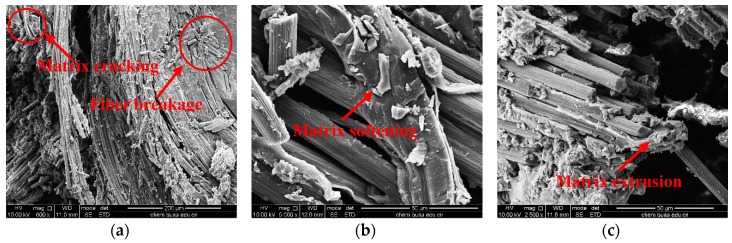

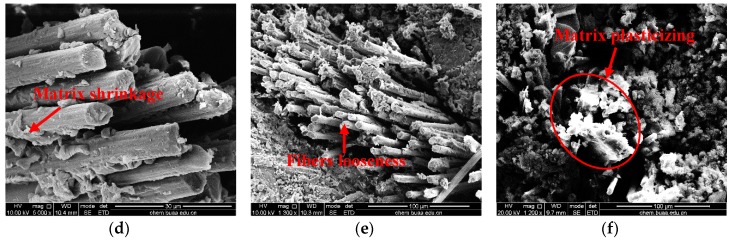
SEM photographs of specimens with 32° braiding angle at different temperatures: (**a**) 25 °C, (**b**) 60 °C, (**c**) 90 °C, (**d**) 120 °C, (**e**) 150 °C, and (**f**) 180 °C.

Microscopic morphologies in Figure 10 further explicit the damage and breakage of 32° specimens at different temperatures. The interior broken mode could be more clearly observed for specimens at 25 °C and 60 °C due to serious cracks which resulted in the specimens being more easily divided into parts. However, for 21° specimens, the structure remained as a whole and the morphologies were all about the thickness direction. At 25 °C, the material damage resulted from fiber and matrix breakage, while the fiber still bonded well with the matrix similar with 21° specimens. Fibers are still restrained tightly among yarns due to the adding of axial yarns. Meanwhile, the fiber bundles still maintained in a certain radian due to their brittle fracture characteristics. At 60 °C and 90 °C, softening of the matrix and the extrusion phenomenon occurred due to the high temperature. Apart from some brittle fibers, most fibers still bonded well with the matrix. At 120 °C, matrix shrinkage at high temperature induced fiber bundles to become loose, and interface debonding between fibers and matrix was obvious, which was similar with the 21° specimen. At 150 °C, axial yarns pulled out and became loose, which was different from integrate fiber bundles pullout of the 21° specimen, since the stress of 32° specimens was mainly carried by fibers and matrix together, and the failure of matrix had a greater effect on the structure of composites. Plasticization and shrinkage of the matrix simultaneously led to more cracks among fibers. At 180 °C, the surface matrix content of fiber was less because of the high plasticizing of matrix when the temperature was above T_g_ for 32° specimens; large braiding angle creates space conditions for matrix reservation, which is different from that of 21° specimen.

Therefore, the 3D five-directional braided composites still kept as a whole after out-of-plane compression, which is superior to other composites with serious delaminating phenomenon, as discussed in the literature [36,37,38,39]. In addition, for both 21° and 32° composites, large 45° shear cracks became gradually obscure and small cracks became more and more prevalent along fiber bundles with the rising temperature due to the stress that could not be effectively transmitted to fibers because of matrix softening. Moreover, the axial yarns prevented the structure from being destroyed during the crack propagation process. Finally, for 21° composite, the stress was mainly carried by the fibers, and the axial yarns inhibited the fiber bundles deformation with a large angle during compression; with regards to 32° composite, the stress was carried by fibers and matrix together, and secondary cracks could be observed along 45° shear cracks resulting from crack propagation.

## 4. Conclusions

3D five-directional braided carbon/epoxy composites with different braiding angles were elaborated successfully. The axial yarns effectively improved stress–strain curves and compression properties compared with four-directional composites. Compression stress–strain curves of composites at different temperature points underwent a linear increase. The load dropped dramatically at 25–90 °C before it increased again compared with that at 120–180 °C. Furthermore, the curve of large braiding angle composite dropped more significantly when compared with a small braiding angle composite after reaching its peak. Both temperature and braiding angle were important parameters affecting the compression properties of composites and the properties decreased with the rising temperature for both 21° and 32° composites. Moreover, the small braiding angle composite had higher properties than the large braiding angle composite at each temperature point.

The damage for composites with different braiding angles occurred in the braiding direction. At 25–90 °C, the damage was symmetric ±45° shear crack for 21° composite and thorough 45° shear crack for 32° composite at 25–90 °C which resulted from fiber, fiber bundles, and matrix fracture; additionally, the axial yarns could act as additional objects to bear the capacity. At 120–150 °C, the failure was caused by looseness in braiding yarns and axial yarns and cracks propagation along fiber bundles due to the matrix softening and plasticizing. The 45° shear crack became obscure, and fiber squeezing deformation and fiber bundle looseness became increasingly serious due to the softness of the matrix. At 180 °C, small cracks along fiber bundles were obvious owing to further plasticization and decomposition of matrix. Furthermore, the small braiding angle composite was more solid and firm and the damage for the large braiding angle composite became more serious.

## Figures and Tables

**Table 1:** The details of out-plane compression specimens.

Sample No.	T (°C)	Braiding Angle α (°)	Fiber Volume Fraction (%)	Length (mm)	Width (mm)	Thickness (mm)	Weight (g)	Density (g·cm^−3^)
3B5D21-1	25	21	49.27%	9.64	9.57	8.13	1.15	1.53
3B5D21-2	60	21	49.27%	9.59	9.50	8.05	1.12	1.53
3B5D21-3	90	21	49.27%	10.04	9.53	8.05	1.16	1.51
3B5D21-4	120	21	49.27%	9.52	9.55	8.07	1.12	1.53
3B5D21-5	150	21	49.27%	9.77	9.53	8.19	1.15	1.51
3B5D21-6	180	21	49.27%	9.65	9.54	8.17	1.13	1.50
Mean	–	–	–	9.70	9.54	8.11	1.14	1.52
S*	–	–	–	0.19	0.02	0.06	0.02	0.01
3B5D32-1	25	32	48.84%	9.83	9.55	8.83	1.19	1.44
3B5D32-2	60	32	48.84%	9.62	9.57	8.91	1.18	1.44
3B5D32-3	90	32	48.84%	9.64	9.52	8.88	1.17	1.44
3B5D32-4	120	32	48.84%	9.65	9.51	8.89	1.16	1.42
3B5D32-5	150	32	48.84%	9.68	9.57	8.82	1.19	1.46
3B5D32-6	180	32	48.84%	9.55	9.41	8.87	1.16	1.46
Mean	–	–	–	9.66	9.52	8.87	1.18	1.44
S*	–	–	–	0.09	0.06	0.03	0.01	0.02

Note: S*, standard deviation.

**Table 2:** The strength and modulus of specimens.

Temperature (°C)	Strength (MPa)		Modulus (GPa)	
	3B5D21	3B5D32	3B5D21	3B5D32
25	305.82	285.94	3.27	2.94
60	301.04	283.21	2.78	2.62
90	283.31	277.34	2.63	2.49
120	260.00	252.07	2.42	2.23
150	240.51	220.98	2.06	1.84
180	221.91	207.56	1.56	1.51
Mean	268.77	254.52	2.45	2.27
S	33.75	33.67	0.59	0.53
